# New light on the horizon of Alzheimer's disease

**DOI:** 10.1093/nsr/nwaa008

**Published:** 2020-05-25

**Authors:** Mu-ming Poo

**Affiliations:** 1 Mu-ming Poo Scientific Director; 2 CAS Center for Excellence for Brain Science and Intelligence Technology, China; 3 Executive Editor-in-Chief of NSR

The beginning of the year 2020 is marked by a groundbreaking event in drug development in China. A first-in-class drug for Alzheimer's disease (AD), GV-971, received conditional approval by the China Food and Drug Administration for the Chinese market. China now has at least 10 million patients with diagnosed AD, afflicting almost one-third of the population aged 90–94 years. There is increasing demand for new AD drugs, as the societal burden caused by this devastating disease is becoming a major healthcare issue in all aging societies. This urgency is reflected by the conditional approval of the drug, a widely used method by drug regulatory agencies in advanced countries to expedite promising new medicines to the market and facilitate early patient access, before long-term assessment of the treatment is available.

According to the World Health Organization, brain disorders, including neurological and psychiatric diseases, now impose a higher societal burden than all other diseases, including cancer and cardiovascular diseases. However, development of drugs for treating brain disorders has been highly disappointing, despite huge investment by major pharmaceutical companies. The causes of failure include the difficulty in understanding pathogenic mechanisms underlying many brain disorders, hence inappropriate identification of drug targets, and the inadequacy of animal models of brain disorders for pre-clinical efficacy test of lead compounds. What is different in the case of GV-971?

GV-971 is an oligosaccharide with a complex structure isolated from seaweed. According to a recent paper published in *Cell Research* by its lead inventor Meiyu Geng and coworkers, GV-971 is capable of restoring the normal pattern of gut microbiota, a large group of microorganisms living in the gut symbiotically with the host. There is now increasing evidence for a link between gut dysbiosis and inflammation in the brain which can cause many brain disorders, including AD, Parkinson's disease, and autism spectrum disorders. The chief culprit of neuroinflammation is a special group of immune cells in the brain. Geng and coworkers showed in a mouse model of AD that the abnormal pattern of gut bacteria is correlated with increased differentiation and proliferation of peripheral T cells, elevated infiltration of a pro-inflammatory subtype of T cells into the brain and activation of a resident pro-inflammatory subtype of microglia, as well as the appearance of AD pathological markers such as amyloid-β (Aβ) plaques. Treatment with GV-971 restores the normal gut microbiota, reduces the level of inflammatory cells and Aβ plaques in the brain, and ameliorates cognitive impairment of the AD model mice.

Unlike previous approaches of targeting at the generation or the existing level of AD pathologic markers, GV-971’s efficacy may be attributed to the fact that it acts upstream at the origin of pathogenesis, i.e., the abnormal gut microbiota that causes neuroinflammation, and thus is capable of slowing AD progression in patients with early and mid-stage AD. In this light, GV-971 may prove to be effective in treating other neurodegenerative diseases besides AD. On the other hand, there are many mechanistic issues to be clarified – how GV-971 exerts preferential actions on the proliferation of different species of gut bacteria, how abnormal gut dysbiosis causes peripheral and central inflammatory responses, and whether GV-971 acts directly in the brain to reduce neuroinflammation and Aβ plaque formation. These issues are intimately associated with gut–brain interaction, a brand new frontier in biomedical research.

Research on biological mechanisms could help to advance drug development by identifying drug targets. Yet many effective drugs, Chinese herb medicines in particular, have long been used without clear mechanistic understanding. An interesting lesson from GV-971 is that the idea of using natural oligosaccharides, guided by the broad scientific concepts of gut microbiome and neuroinflammation, together with a utilitarian perspective, may have achieved a milestone in AD drug development, surpassing decade-long attempts in developing drugs based on mechanistic identification of drug targets.

Development of a first-in-class drug is a long process that requires vision, courage, and persistence of both researchers and investors. It has taken more than two decades for GV-971 to reach the market – from the inception of the idea to pre-clinical studies on model animals, followed by long and costly clinical trials, all amid the looming prospect of failure. For many biomedical researchers in China, translational effort has been overshadowed by the desire for fast publications that bring immediate recognition and reward. To most Chinese pharmaceutical investors, developing new drugs is much less likely to yield profit than making imitation drugs. The success of GV-971 demonstrates that innovative drug development, like all forms of innovation, requires the audacity of researchers and investors in exploring the road not taken. With the persistent call and support for innovation from the government, we look forward to a stream of first-in-class drugs appearing in China, particularly those treating brain disorders.

